# Poly-ϵ-caprolactone scaffold as staple-line reinforcement of rectal anastomosis: an experimental piglet study

**DOI:** 10.1186/s12876-024-03202-1

**Published:** 2024-03-15

**Authors:** Laura Lovisa Køtlum Petersen, Martin Dennis Dursun, Gunvor Madsen, Dang Quang Svend Le, Sören Möller, Niels Qvist, Mark Bremholm Ellebæk

**Affiliations:** 1 Research Unit of Surgery, Odense University Hospital, Odense, Denmark; 2 University of Southern Denmark, Odense, Denmark; 3 Research Unit of Pathology, Odense University Hospital, Odense, Denmark; 4 Department of Clinical Medicine, Aarhus University, Aarhus, Denmark; 5 Open Patient data Explorative Network, Department of Clinical Research, Odense University Hospital and Research unit OPEN, University of Southern Denmark, Odense, Denmark

**Keywords:** Rectal anastomosis, Poly-epsilon-caprolactone, Staple-line, Tensile strength, Anastomotic wound healing, Anastomotic leakage

## Abstract

**Purpose:**

Rectal anastomoses have a persisting high incidence of anastomotic leakage. This study aimed to assess whether the use of a poly-ϵ-caprolactone (PCL) scaffold as reinforcement of a circular stapled rectal anastomosis could increase tensile strength and improve healing compared to a control in a piglet model.

**Method:**

Twenty weaned female piglets received a stapled rectal anastomosis and were randomised to either reinforcement with PCL scaffold (intervention) or no reinforcement (control). On postoperative day five the anastomosis was subjected to a tensile strength test followed by a histological examination to evaluate the wound healing according to the Verhofstad scoring.

**Results:**

The tensile strength test showed no significant difference between the two groups, but histological evaluation revealed significant impaired wound healing in the intervention group.

**Conclusion:**

The incorporation of a PCL scaffold into a circular stapled rectal anastomosis did not increase anastomotic tensile strength in piglets and indicated an impaired histologically assessed wound healing.

## Introduction

Despite ongoing development in surgical techniques, anastomotic leakage (AL) persists as a major postoperative complication following surgery such as resections on the rectum irrespective of the underlying disease. AL is associated with increased morbidity and mortality in addition to longer hospitalisation and considerably poorer long-term oncological outcome in rectal cancer patients [1, 2]. The risk of leakage varies from 6 to 11.6% [3–6] but depending on the definition of AL up to 20% risk has been described [7–9].

The cause of AL is multifactorial, it differs from other types of wound healing and is not very well understood [10, 11]. Potential causes may be impaired wound healing, anastomotic tension, tissue hypoperfusion and infection but other biochemical factors are not well understood [12]. Risk factors include male gender, current smoking, excessive alcohol consumption, obesity, low level anastomoses and advanced tumour stage [3, 6, 8].

Several attempts have been made to reinforce the stapled anastomosis to reduce the occurrence of AL, including various biodegradable and bioabsorbable materials for staple-line reinforcement, but have failed to show convincing results [13–16]. Poly-ϵ-caprolactone (PCL) is a biodegradable polymer which degradation kinetics may be tailored and is easy to mould. The material has been investigated as reinforcement of vascular anastomoses and has been successfully used to administer active agents for promoting bone growth [17, 18].

Previous studies conducted at our institution have shown that incorporation of a specially designed PCL scaffold into a circular stapler was able to significantly increase the anastomotic tensile strength in the small intestine at postoperative day five in a piglet model [19]. The incorporation did not increase the risk of stenosis evaluated on postoperative day 30 [20]. To our knowledge, no studies have investigated the use of PCL scaffolds in the reinforcement of stapled rectal anastomoses.

The objective of this experimental piglet study was to investigate the effect of a PCL scaffold incorporated into the staple-line of a circular stapled rectal anastomosis compared to a control anastomosis without PCL. The primary outcome was anastomotic tensile strength on postoperative day five with histological evaluation of the wound healing as the secondary outcome.

## Materials and methods

### Study design

In this open labelled randomised study, piglets underwent an end-to-end circular stapled anastomosis of the rectum. In the intervention group, a PCL scaffold was incorporated into the staple-line while the control group received a conventional circular stapled anastomosis. Five days postoperatively, the animals were euthanised, and all anastomoses were subjected to a tensile strength test and subsequent histologic examination.

### Ethical statement

This study follows the ARRIVE guidelines for reporting of animal research studies. Measures were taken to ensure minimal suffering of test animals during the trial period. This project was approved by the Danish Animal Experiments Inspectorate (2018-15-0201-01583).

### Animals

Twenty-two weaned female Landrace/Yorkshire piglets were initially included in the study. The animals were housed at an approved facility and were acclimatised for 1 week prior to trial start. The piglets had free access to water and were fed twice a day with standard pig chow.

A medical record was obtained for each animal including all medicine administered, pre- and postoperative monitoring and surgical course. To ensure animal welfare, the piglets were observed every 6th hour in the first 24 h postoperatively, and afterwards regularly during daytime and once at night. Each animal was weighed prior to surgery and at study completion on postoperative day five.

### Randomisation

Randomisation was done using Research Randomizer® 4.0 utilising a block design with 5 blocks of 4 animals and 1 block of 2, the succession of which was also randomised. The randomisation was concealed to the investigators until the start of surgery. The pathologist evaluating the histology was blinded to the randomisation.

### Anaesthesia

The piglets were pre-anaesthetised with a combination of 0.25 mg/kg midazolam (Hameln Pharma Plus GmbH, Hameln, Germany), 0.03 mg/kg medetomidine (Cepetor®, ScanVet Animal Health A/S, Fredensborg, Denmark), 5 mg/kg ketamine (Ketaminol®, MSD Animal Health A/S, Copenhagen, Denmark) and 0.2 mg/kg butorphanol (Butomidor®, Salfarm Danmark A/S, Kolding, Denmark) administered intramuscularly.

Anaesthesia was induced with 5 mg/kg propofol and maintained with a continuous infusion of 15 mg/kg/hour propofol (B. Braun Melsungen AG, Melsungen, Germany) and 25–50 µg/kg/hour fentanyl (B. Braun Melsungen AG, Melsungen, Germany) administered intravenously. The piglets were intubated and ventilated with a respiratory frequency and a tidal volume adjusted to an end tidal CO_2_ of approximately 7 kPa using a Siesta i TS Anaesthesia Machine (Dameca, Rødovre, Denmark). Blood pressure, electrocardiogram, heart rate, oxygen saturation and body temperature were monitored continuously during operation.

Antibiotic treatment included 20 mg/kg amoxicillin (Curamox ® Prolongatum, Boehringer Ingelheim Danmark A/S, Copenhagen, Denmark) administered intramuscularly prior to operation and 20 mg/kg metronidazole (B. Braun Melsungen AG, Melsungen, Germany) administered intravenously during operation.

Postoperative analgesia consisted of 0.03 mg/kg buprenorphine (Bupaq ® Multidose, Salfarm Danmark A/S, Kolding, Denmark) administered by intramuscular injection every 6th hour until a sufficient analgesic effect of fentanyl transdermal patch (Matrifen® 37 µg/hour, Takeda Pharma A/S, Taastrup, Denmark) was achieved, based on clinical evaluation of the piglets.

At re-laparotomy, all piglets were sedated using a Zoletil mix; a mixture of 25 mg/ml zolazepam/tiletamine (Zoletil® 50 vet, Virbac Danmark A/S, Kolding, Denmark), 10.9 mg/ml xylazine (Rompun® Vet, 20 mg/ml, Bayer Animal Health GmbH, Leverkusen, Germany), 10.9 mg/ml ketamine (Ketaminol® Vet, 100 mg/ml, MSD Animal Health A/S, Copenhagen, Denmark), 1.7 mg/ml butorphanol (Butomidor® Vet, 10 mg/ml, Salfarm Danmark A/S, Kolding, Denmark) and 1.7 mg/ml methadone hydrochloride (Comfortan® Vet, 10 mg/ml, Eurovet Animal Health B.V., Bladel, Netherland) administered intravenously at a dose of 0.13 ml/kg. One ml/20kg of Zoletil mix was administered every 20–30 min as needed to ensure adequate sedation evaluated by continuous reflex monitoring. Due to the lighter sedation, the piglets maintained spontaneous ventilation throughout the procedure.

At study completion all piglets were euthanised using 140 mg/kg pentobarbital sodium (Exagon® Vet, Richter Pharma AG, Wels, Austria) administered intravenously.

### Scaffold material

**Fig. 1:**
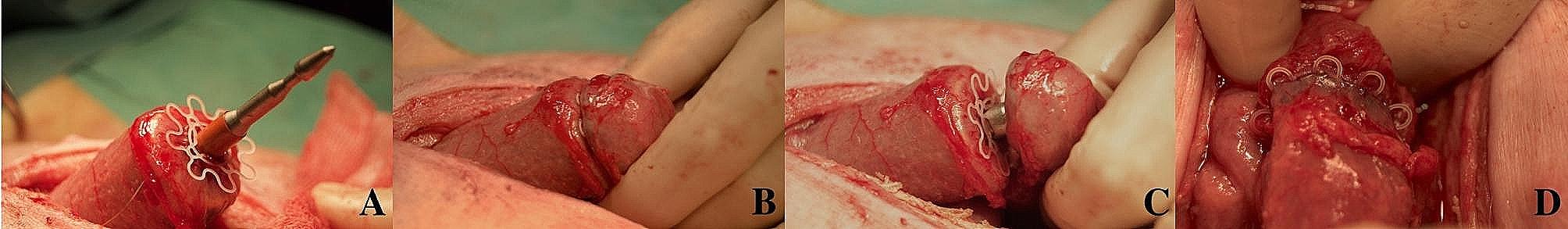
Rectal anastomosis with a Poly-ϵ-caprolactone (PCL) scaffold. **(A)** Trocar with scaffold. **(B)** Trocar and anvil connected. The trocar is halfway retracted, and the scaffold is in place. **(C)** Before activation of the stapler. **(D)** Stapled anastomosis with PCL scaffold reinforcement

The scaffold used in this study was manufactured from 80 kDa poly-ϵ-caprolactone (Sigma-Aldrich, Denmark) and designed using Autodesk Inventor® (version 2016; California, USA) and a 3D printer (nScrypt 3D300TE 3D, nScrypt, FL). The scaffold was shaped as a double undulating ring with eight flexible loops with an outer diameter of 22.7 mm (Fig. 1A). The scaffold design fit onto the stapler trocar with alignment matching the staple pattern (Fig. 1D) resulting in uncompromised staple-line continuity and flexibility while providing reinforcement. After firing the stapler, the inner ring of the scaffold was cut, leaving only the outer ring incorporated in the anastomotic line. Prior to incorporation, the PCL scaffold was sterilised in 10% hydrogen peroxide for 30 min and subsequently rinsed repeatedly in sterile water [21].

### Surgical procedure

In preparation, the rectum was emptied from faecal content using a syringe with tap water. The rectum was exposed through a lower midline laparotomy and the rectum was divided at the most oral region using a straight scissor. A purse-string suture monocryl^TM^*Plus*® 4 − 0 (Ethicon, Johnson & Johnson, Diegem, Belgium) was placed in the muscular layer of both bowel ends of the end-to-end anastomosis. The anvil of the circular stapler, size 21 (Ethicon, Johnson & Johnson, Birkerød, Denmark), was placed in the proximal end and the purse-string suture was tied. Afterwards, the stapler was introduced through the anal canal and the distal purse-string suture was tied around the trocar. In the intervention group the PCL scaffold was placed on the trocar (Fig. 1A) prior to tightening and activation of the stapler (Fig. 1B-C). The anastomosis integrity was tested by an air leak test as previously suggested for colorectal anastomoses [22]. At any sign of a defect during testing this was allowed to be corrected with a single suture using monocryl^TM^*Plus*® 4 − 0.

The abdominal fascia was closed with a running suture using 0 PDS^TM^*Plus*® (Ethicon, Johnson & Johnson, Diegem Belgium). The skin was closed with a running intracutaneous suture using monocryl^TM^*Plus*® 2 − 0 (Ethicon, Johnson & Johnson, Diegem, Belgium) and sealed with a liquid bandage (OPSITE spray, Smith&Nephew Medical Limited, 101 Hessle Rd, England).

At study termination a re-laparotomy was performed. The anastomosis was identified and carefully freed from adhesions. Resection was made at approximately 5 cm on either side of the anastomosis and subsequently mounted in the testing machine.

### Maximum Tensile Strength (MATS) test

For the tensile strength a LF Plus testing machine (Lloyds Instruments, Fareham, UK) equipped with an XLC 100 N loadcell (Lloyds Instruments, Fareham, UK) was used. The resected rectum was clamped at each bowel end with a 60 mm gap between the clamps with the anastomotic line in the middle. The test was performed within eight minutes following resection and with a constant deformation rate of 15 mm/min until transmural rupture occurred. The force applied was measured at two time points; when a tear became visible in the serosa (MATS-1) and when a transmural rupture appeared (MATS-2). Finally, the location of the rupture within or outside the anastomosis was noted.

### Macroscopic examination

All anastomoses were examined and macroscopic findings such as pseudodiverticulosis, abscesses, visible leakage, signs of ileus and stenosis were noted. Adhesions scores were determined with zero indicating no adhesions or adhesions not involving the staple-line and 1 indicating adhesions involving the staple-line.

### Histological examination

After removal of the staples, tissue samples including the anastomosis were placed in a 10% formaldehyde solution for a minimum of 48 h. Tissue samples were then embedded in paraffin, cut to 4 μm slices and subsequently stained with haematoxylin and eosin (HE) for evaluation of necrosis, polymorphonuclear cells, lymphocytes, macrophages, oedema, and mucosal epithelium. The evaluation of smooth muscle growth was performed with alpha smooth muscle actin (α-SMA) and desmin staining. All tissue samples were examined by the same pathologist and scored according to Verhofstad Scoring system [23]. Each parameter was assigned a score of 0–3; the lowest score reflecting normal tissue and optimal healing. A mean value was calculated for each parameter in the control and intervention group.

Collagen content was evaluated with picrosirius red staining as a measure of healing and strength in the tissue.

### Power calculation and statistical analysis

In this study, an increase of 20% in tensile strength was considered a clinically significant difference. A previous study [17] of the PCL scaffold in small intestinal anastomoses reported MATS-2 in the intervention group of 15.1 N (± 3.3 N) and 11.8 N (± 4.3 N) in the control group (*p* = 0.01) corresponding to an increase of 22.1%. Based on this study with a power of 80% of detecting the chosen significant increase of 20% and a 0.05 risk of a type I error (α), the sample size calculation yielded a total of 20 anastomoses needed for the final analysis. To account for expected mortality or complications of 10%, 22 piglets needed to be included in the study. Throughout the study a *p*-value < 0.05 was regarded as statistically significant and statistical analyses were performed using Stata/IC (version 16.1; Texas, USA). Mean values of MATS-1 and − 2, weight change, as well as collagen percentage were compared using a t-test, and *p*-value as well as 95% confidence intervals of differences are reported. The ordinal scores of individual parameters in the Verhofstad score, as well as the total score, were compared by non-parametric Wilcoxon sum rank test. Adherence of the anastomoses were evaluated by Fisher’s exact test.

## Results

Twenty-two piglets with a mean weight of 20.2 kg ranging from 17.1 to 22.1 kg were included in the study (Table 1). Two piglets were euthanised prematurely during surgery: One due to surgical complications and one because of severe intra-abdominal scarring from preceding peritonitis. Both piglets were randomised to the intervention group leaving nine pigs in the intervention group and 11 in the control group for analysis. In three cases a minor defect in the anastomosis following stapling was closed with single sutures: one in the intervention-, and two in the control group. No visible or clinical AL occurred in any piglet.

**Table 1:** Maximum tensile strength including frequencies of intra-anastomotic rupture, observational data regarding the test animals, macroscopic and histological findings divided into the interventional group and the control group. All results are reported as mean (± standard deviation, SD) unless stated otherwise

Parameters	PCL scaffold anastomosis	Control anastomosis	*P*-value for difference
Number of test animals included	9	11	
Maximum Tensile Strength (MATS) (Newton)			
MATS-1 MATS-2	18.43 (± 8.31) 18.86 (± 7.81)	17.78 (± 3.67) 21.73 (± 8.48)	0.817 0.446
Intra-anastomotic rupture (number)	0	2	0.479
Weight (kg)			
Preoperative Postoperative day 5 Weight gain	20.45 (± 1.04) 23.14 (± 1.42) 2.84 (± 0.6)	19.87 (± 1.53) 22.05 (± 1.40) 2.17 (± 0.41)	0.315 0.099 **0.009**
Adhesion score (0–1)	0.67 (± 0.5)	0.55 (± 0.52)	0.828
Histological parameters by Verhofstad score (0–3)			
Necrosis Polymorphonuclear cells Lymphocytes Macrophages Oedema Mucosal epithelium repair Submucosal-muscular layer repair Total Verhofstad score Collagen, mean %	0.56 (± 0.53) 2 (± 0.71) 0.67 (± 0.5) 0 1.56 (± 0.53) 3 (± 0) 1.56 (± 0.53) 9.3 (± 1.73) 36.1 (± 12.44)	0.4 (± 0.51) 1.7 (± 0.48) 0.5 (± 0.53) 0 0.9 (± 0.74) 2.6 (± 0.52) 0.91 (± 0.83) 6.9 (± 2.23) 44.1 (± 21.43)	0.509 0.316 0.475 1.000 **0.050** **0.038** 0.073 **0.025** 0.337

### Maximum Tensile Strength (MATS) test

Incorporation of the PCL scaffold in the anastomosis did not significantly improve the maximum tensile strength compared to the control anastomosis of the rectum. MATS-1 tended to be higher in the interventional group with a mean difference of 0.65 N (95% CI, -5.18 to 6.49) while MATS-2 tended to be higher in the control group with a mean difference of 2.87 N (95% CI, -4.87 to 10.60). Intra-anastomotic rupture during the MATS test occurred in two anastomoses (10%) in the control group and none in the intervention group (Table 1).

### Macroscopic findings and histological examination

No pseudo-diverticulosis, intra-abdominal abscess, macroscopic signs of stenosis or ileus was observed in this study.

The mean total Verhofstad score was significantly greater in the intervention group (Table 1). The mean score was 9.3 ± 1.73 in the intervention group compared to 6.9 ± 2.23 in the control group. Analysing the individual parameters, the mean value was higher in the intervention group in all parameters but only significantly higher for mucosal epithelium repair and oedema.

## Discussion

This study did not reveal a significant difference between the use of a PCL scaffold for reinforcement of the staple-line in end-to-end rectal anastomotic compared to no scaffold regarding anastomotic strength evaluated by the tensile strength test on postoperative day five. Intra-anastomotic rupture during tensile strength test occurred in only two piglets in the control group as opposed to none in the intervention group. No piglets developed AL during the study. Histologic examination showed a significantly lower Verhofstad score in the control group, which indicated differences in wound heling between the two groups. Some parameters showed superiority in wound healing and others inferiority in the intervention group.

Many different methods for reinforcements of rectal anastomoses have been suggested. Recently both external and internal sutured reinforcement of a stapled low rectal anastomosis in humans have been described. External suturing resulted in a low frequency of AL, but there was no control group [24]. With an internal suturing of the staple-line the rate of AL without a diverting stoma was the same as conventional anastomosis with a diverting stoma, indicating that the internal suturing enforcement may omit the necessity of a diverting stoma [25]. Additionally, injection of stem cells in the staple-line to promote wound heling is an interesting which has been described in animal experimental studies but not in a clinical setting [26]. A recent review concluded that no single method of anastomotic reinforcement in colorectal anastomosis has improved the risk of AL [27].

Only a few studies have evaluated the use of a staple-line reinforcement approach comparable to this current study. Fajardo et al. [10] investigated a polymeric bioabsorbable SeamGuard (BSG) incorporated into the staple-line of rectal anastomoses in a piglet model (*n* = 22). The BSG reinforcement was also applied directly onto the stapler anvil and cartridge. The method was found to be safe although it exhibited no clear advantages or significant difference in burst pressure compared to no BSG on postoperative day 14. A slight increase in infiltration of inflammatory cells was observed in the BSG group however there was no difference of elastin or collagen concluding that the healing was not compromised.

The use of bioabsorbable staple-line reinforcement (BSLR) of rectal anastomoses in patients has only been reported in two studies. Senagore et al. studied the effect of a synthetic BSLR compared to no reinforcement in a randomised study in 258 patients receiving circular stapled anastomosis < 10 cm from the anal verge [14]. In the study, 85% of patients received faecal diversion at some point within the follow-up of 4–12 weeks or up to 6 months in case of adjuvant chemotherapy but before ileostomy takedown. The study found no significant reduction in AL rate assessed either by endoscopy or contrast enema. Placer et al. investigated the same BSLR with 30 days follow-up (*n* = 302) and failed to show any significant reduction in postoperative complications such as leaks, bleeding or stenosis [15]. The concept of BSLR/BSG is largely comparable to our approach, although the PCL scaffold exhibits some advantages as it, unlike the BSLR/BSG, is mounted after insertion of the stapler and introduction of the spear right before stapling of the anastomosis, thus minimising the risk of the scaffold dismounting as well as unnecessary contamination of the staple-line.

As alternatives to staple-line reinforcement of rectal anastomoses, endoluminal protection and external reinforcement have been investigated with limited protective effects. In the C-seal trial [28], endoluminal protection of the rectal anastomosis was investigated in a clinical randomised study (*n* = 402) using a circular stapler and a biodegradable polymeric sheet attached to the staple-line reinforcement which was covering the anastomosis from luminal content. The study failed to show any differences in the frequency of AL compared to no protection. On the other hand, external coating of colonic anastomoses including fibrin sealant, omental pedicle graft and hyaluronic acid/carboxymethylcellulose has been evaluated in a review including both animal and human studies [29]. In humans, only fibrin sealant has shown positive, although not significant results when using fibrin sealant compared to no sealant when performing rectal anastomoses in 233 patients with rectal cancer [30]. Wenger et al. found the incidence of AL at postoperative day nine to be 20% in the control group and 5% in the intervention group receiving fibrin sealant as reinforcement of a circular stapled colorectal anastomosis in pigs (*n* = 40) [31]. Using the same method, no cases of AL were found in the intervention group at 21 days follow-up compared to 20% in the control group [32]. In contrast to these methods, the PCL scaffold is aimed at increasing the tensile strength of the anastomosis in the beginning of wound healing while not compromising the healing process. Assessment of wound healing using the Verhofstad score showed signs of inferior healing in the intervention group compared to the control group in this current study. Incorporation of the PCL scaffold or the process of applying it might have contributed to impaired healing although measures were taken to disinfect and rinse the scaffold before stapling and non-inferior healing has been shown previously in the small intestine [19, 20]. Furthermore, removal of staples and PCL scaffolds from the anastomoses prior to histological preparation led to an unknown degree of tissue damage and random errors, however, it enabled blinding of the pathologist and ensured uncompromised specimen for histology. Additionally, histologic grading using Verhofstad score was originally designed for rats [23] and may be suboptimal for evaluating healing in the porcine rectum at this duration of wound healing.

Previously, an equivalent PCL scaffold was tested in the small intestine in piglets which showed an increase in tensile strength of over 20% compared to anastomoses without PCL scaffold [19]. Similar effects of the PCL scaffold were not observed in the rectum although tested on the same equipment and similar piglets. Rectal wall thickness and structure could be an attributing factor to the PCL scaffold’s apparent inability to increase the strength in the intervention group, as the staplers were fitted tightly into the tissue in both groups. Supporting this hypothesis, the tensile strength of the rectum intestinal wall of the control anastomoses is approximately 40% higher than the control anastomoses of the small intestine in Larsen et al. [19]. The measuring of tensile strength by visual presentation is made difficult due to the relatively thick intestinal wall. Although a defect in serosa was visually detected, it was not clearly visible on the load over time graph. Although this method of evaluating the anastomotic strength has been widely used, it is a suboptimal assessment of the anastomosis itself. It is therefore worth considering, if other assessment methods could produce different results of the anastomotic strength.

During the tensile strength test, intra-anastomotic rupture occurred only twice, both in the control group. Thus, only in these anastomoses, the true anastomotic strength was directly reflected by the test. This small percentage of intra-anastomotic rupture is a known concern when investigating colorectal anastomoses in animal models and results in uncertain conclusiveness of the studies regarding anastomotic strength [13, 16]. This may be the result of inability of the test to measure the anastomotic strength beyond the strength of the surrounding native tissue.

The strength test was performed with a 60 mm gap between the clamps which might have contributed to higher rate of extra-anastomotic rupture due to excess tissue being stretched during the test and thus presenting a larger surface for a potential rupture. A smaller gap of e.g.10 mm, could possibly prevent the large number of extra-anastomotic ruptures, as less tissue is stretched during the test, as used by Kjaer et al. [33].

Ikeuchi et al. investigated the correlation between tensile strength and bursting pressure in intestinal anastomoses on a colonic rat model to test if the two widely used measures were equal [34]. They found significant correlations between the methods only in the proliferation phase of healing after day five (days 5–12) but stated that tensile strength is also a strong indicator of suture holding power. Whether tensile strength or burst pressure is superior depends on the individual setting it is being used in but both methods are valid surrogate measures and strengthened by the concomitant histological evaluation [35]. In this present study, tensile strength was chosen, as it is considered a valid method for evaluating anastomotic strength at this relatively short follow-up [36]. However, it must be considered that the tensile strength may not be directly transferable to the true integrity of the anastomosis due to AL not necessarily being caused by longitudinal tension [37]. Consequently, there is a risk of bias due to the inability of tensile strength test to determine the potential strength addition that is not related to direct longitudinal force. No consensus has been reached regarding the ideal postoperative day for investigation of intestinal anastomoses. Postoperative day five was chosen for evaluation of the anastomosis matching the initial proliferation phase where ingrowth of fibroblasts and collagen synthesis has begun [10]. Considering that the median postoperative time to diagnosis of AL in patients is around seven days [38] and the fact that the diagnosis of AL often is delayed [39], this follow-up was deemed a reasonable timing of evaluation. Tensile strength as a surrogate endpoint for AL is regarded as a suitable alternative to reporting the incidence of AL during a longer follow-up due to the potentially prolonged time before clinical presentation of AL and use of a minimum number of animals. A recognized weakness of the present study is that the experiments were performed on normal rectal tissue. In the clinical situation, structural changes in tissue related to disease or preoperative radiochemistry might result in a different outcome.

In conclusion, the PCL scaffold did not increase anastomotic strength in rectal anastomoses and the histological scoring of wound healing showed changes indicating both improved and impaired wound healing. The fact that there was no ruptures in the anastomotic line in the intervention group indicated that the incorporation of a scaffold in the staple-line was feasible. Whether this may be transferred to the clinical setting is unknown. Further investigations using different evaluation methods are needed to determine the long-term effects.

## Data Availability

Original data is available at specific request to the corresponding author.

## References

[1] Kube R, Mroczkowski P, Granowski D, Benedix F, Sahm M, Schmidt U (2010). Anastomotic leakage after colon cancer surgery: a predictor of significant morbidity and hospital mortality, and diminished tumour-free survival. Eur J Surg Oncol.

[2] Karim A, Cubas V, Zaman S, Khan S, Patel H, Waterland P (2020). Anastomotic leak and cancer-specific outcomes after curative rectal cancer surgery: a systematic review and meta-analysis. Tech Coloproctol.

[3] Bertelsen CA, Andreasen AH, Jorgensen T, Harling H, Danish Colorectal Cancer G (2010). Anastomotic leakage after anterior resection for rectal cancer: risk factors. Colorectal Dis.

[4] Paun BC, Cassie S, MacLean AR, Dixon E, Buie WD (2010). Postoperative complications following surgery for rectal cancer. Ann Surg.

[5] Krarup PM, Jorgensen LN, Andreasen AH, Harling H (2012). Danish colorectal Cancer G. A nationwide study on anastomotic leakage after colonic cancer surgery. Colorectal Dis.

[6] Eriksen MT, Wibe A, Norstein J, Haffner J, Wiig JN, Norwegian Rectal Cancer G (2005). Anastomotic leakage following routine mesorectal excision for rectal cancer in a national cohort of patients. Colorectal Dis.

[7] Blok RD, Stam R, Westerduin E, Borstlap WaA, Hompes R, Bemelman WA (2018). Impact of an institutional change from routine to highly selective diversion of a low anastomosis after TME for rectal cancer. Eur J Surg Oncol.

[8] McDermott ED, Heeney A, Kelly ME, Steele RJ, Carlson GL, Winter DC (2015). Systematic review of preoperative, intraoperative and postoperative risk factors for colorectal anastomotic leaks. Br J Surg.

[9] Penna M, Hompes R, Arnold S, Wynn G, Austin R, Warusavitarne J (2019). Incidence and risk factors for anastomotic failure in 1594 patients treated by Transanal Total Mesorectal Excision: results from the International TaTME Registry. Ann Surg.

[10] Thompson SK, Chang EY, Jobe BA (2006). Clinical review: Healing in gastrointestinal anastomoses, part I. Microsurgery.

[11] Morgan RB, Shogan BD (2022). The Science of Anastomotic Healing. Semin Colon Rectal Surg.

[12] Lam A, Fleischer B, Alverdy J (2020). The Biology of Anastomotic Healing-the unknown overwhelms the known. J Gastrointest Surg.

[13] Fajardo AD, Amador-Ortiz C, Chun J, Stewart D, Fleshman JW (2012). Evaluation of bioabsorbable seamguard for staple line reinforcement in stapled rectal anastomoses. Surg Innov.

[14] Senagore A, Lane FR, Lee E, Wexner S, Dujovny N, Sklow B (2014). Bioabsorbable staple line reinforcement in restorative proctectomy and anterior resection: a randomized study. Dis Colon Rectum.

[15] Placer C, Enríquez-Navascués JM, Elorza G, Timoteo A, Mugica JA, Borda N (2014). Preventing complications in colorectal anastomosis: results of a randomized controlled trial using bioabsorbable staple line reinforcement for circular stapler. Dis Colon Rectum.

[16] Boersema GSA, Vennix S, Wu Z, Te Lintel Hekkert M, Duncker DJGM, Lam KH (2017). Reinforcement of the colon anastomosis with cyanoacrylate glue: a porcine model. J Surg Res.

[17] Motamedian SR, Hosseinpour S, Ahsaie MG, Khojasteh A (2015). Smart scaffolds in bone tissue engineering: a systematic review of literature. World J Stem Cells.

[18] Schönfeld A, Kabra ZM, Constantinescu M, Bosshardt D, Stoffel MH, Peters K (2017). Binding of indocyanine green in polycaprolactone fibers using blend electrospinning for in vivo laser-assisted vascular anastomosis. Lasers Surg Med.

[19] Larsen KD, Westerholt M, Madsen GI, Le DQS, Qvist N, Ellebæk MB (2019). Poly-ε-caprolactone scaffold for the reinforcement of stapled small intestinal anastomoses: a randomized experimental study. Langenbecks Arch Surg.

[20] Schiellerup NS, Wismann J, Madsen GI, Le DQS, Qvist N, Ellebæk MB (2021). Incorporation of a Poly-ε-Caprolactone Scaffold in a;Circular Stapled End-To-End Small Intestine Anastomosis does not have any adverse effects within 30 days: a study in piglets. Surg Innov.

[21] Sagripanti JL, Bonifacino A (1996). Comparative sporicidal effect of liquid chemical germicides on three medical devices contaminated with spores of Bacillus subtilis. Am J Infect Control.

[22] Wu Z, van de Haar RCJ, Sparreboom CL, Boersema GSA, Li Z, Ji J (2016). Is the intraoperative air leak test effective in the prevention of colorectal anastomotic leakage? A systematic review and meta-analysis. Int J Colorectal Dis.

[23] Verhofstad MH, Lange WP, van der Laak JA, Verhofstad AA, Hendriks T (2001). Microscopic analysis of anastomotic healing in the intestine of normal and diabetic rats. Dis Colon Rectum.

[24] Barrios P, Ramos I, Crusellas O, Sabia D, Mompart S, Martín-Baranera M (2021). Colorectal anastomosis during cytoreductive radical surgery in patients with peritoneal surface malignancies. Validation of a new technique (without stoma) to prevent anastomosis leakage in more than 1000 procedures. Clin Transl Oncol.

[25] Altomare DF, Delrio P, Shelgyn Y, Rybakov E, Vincenti L, De Fazio M (2021). Transanal reinforcement of low rectal anastomosis versus protective ileostomy after total mesorectal excision for rectal cancer. Preliminary results of a randomized clinical trial. Colorectal Dis.

[26] Trébol J, Georgiev-Hristov T, Pascual-Miguelañez I, Guadalajara H, García-Arranz M, García-Olmo D (2022). Stem cell therapy applied for digestive anastomosis: current state and future perspectives. World J Stem Cells.

[27] Uppal A, Pigazzi A (2021). New technologies to prevent anastomotic leak. Clin Colon Rectal Surg.

[28] Bakker IS, Morks AN, Hoedemaker HO, Burgerhof C, Leuvenink JGM, van Praagh HG (2017). Randomized clinical trial of biodegradeable intraluminal sheath to prevent anastomotic leak after stapled colorectal anastomosis. BJS (British J Surgery).

[29] Pommergaard HC, Achiam MP, Rosenberg J (2012). External coating of colonic anastomoses: a systematic review. Int J Colorectal Dis.

[30] Huh JW, Kim HR, Kim YJ (2010). Anastomotic leakage after laparoscopic resection of rectal cancer: the impact of fibrin glue. Am J Surg.

[31] Wenger FA, Szucsik E, Hoinoiu BF, Cimpean AM, Ionac M, Raica M (2015). Circular anastomotic experimental fibrin sealant protection in deep colorectal anastomosis in pigs in a randomized 9-day survival study. Int J Colorectal Dis.

[32] Wenger FA, Szucsik E, Hoinoiu BF, Cimpean AM, Matonick JP, Ionac M (2019). Is circular fibrin sealing of low rectal anastomosis able to prevent leakage in 21-Day Follow-up? Randomized Experimental Trial in pigs. Surg Innov.

[33] Kjaer M, Krarup PM, Olsen DMG, Russel W, Jorgensen LN, Ågren MS. Optimal use of biomechanical methods in research on colonic anastomotic wound healing.

[34] Ikeuchi D, Onodera H, Aung T, Kan S, Kawamoto K, Imamura M (1999). Correlation of Tensile Strength with bursting pressure in the evaluation of intestinal anastomosis. Dig Surg.

[35] Bosmans J, Moossdorff M, Al-Taher M, van Beek L, Derikx JPM, Bouvy ND (2016). International consensus statement regarding the use of animal models for research on anastomoses in the lower gastrointestinal tract. Int J Colorectal Dis.

[36] Christensen H, Langfelt S, Laurberg S (1993). Bursting strength of experimental colonic anastomoses. A methodological study. Eur Surg Res.

[37] Durães L, de C, Durães EFR, Lobato LF, de Oliveira C, de Sousa PG (2013). Correlation between bursting pressure and breaking strength in colonic anastomosis. Acta Cir Bras.

[38] Li YW, Lian P, Huang B, Zheng HT, Wang MH, Gu WL et al. Very Early Colorectal Anastomotic Leakage within 5 Post-operative Days: a More Severe Subtype Needs Relaparatomy. Sci Rep [Internet]. 2017 Jan 13 [cited 2020 Jul 22];7. Available from: https://www.ncbi.nlm.nih.gov/pmc/articles/PMC5233968/. 10.1038/srep39936 PMC5233968 28084305

[39] den Dulk M, Noter SL, Hendriks ER, Brouwers M a., van der Vlies M, Oostenbroek CH et al. RJ,. Improved diagnosis and treatment of anastomotic leakage after colorectal surgery. Eur J Surg Oncol. 2009;35(4):420–6. 10.1016/j.ejso.2008.04.009 18585889

